# Autophagy and Alzheimer’s disease: How far science has to be progressed? − correspondence

**DOI:** 10.1097/MS9.0000000000000156

**Published:** 2023-01-18

**Authors:** Talha B. Emran, Hitesh Chopra, Kuldeep Dhama

**Affiliations:** a Department of Pharmacy, BGC Trust University Bangladesh, Chittagong, Bangladesh; b Department of Pharmacy, Faculty of Allied Health Sciences, Daffodil International University, Dhaka, Bangladesh; c Chitkara College of Pharmacy, Chitkara University, Punjab, India; d Division of Pathology, ICAR-Indian Veterinary Research Institute, Bareilly, Izatnagar, Uttar Pradesh India

Alzheimer’s disease (AD) is the most common neurodegenerative disorder affecting older adults globally^1^. The main clinical symptom of AD is memory loss, which is followed as the disorder advances by abnormalities in cognitive and adaptive functioning. AD can be broadly characterized as familial and sporadic based on several criteria^2^. Early onset AD, commonly referred to as familial AD, is frequently linked to genetic abnormalities, including those in the amyloid precursor protein (APP), presenilin 1, and presenilin 2. Environmental and complicated genetic variables, such as the existence of the epsilon 4 allele in apolipoprotein E, frequently contribute to sporadic AD^3^. In terms of its pathology, AD is defined by the accumulation of toxic tau-enriched neurofibrillary tangles (NFTs) and β-amyloid (Aβ) aggregates in the cerebral cortex and hippocampus of the brain^4^. Disruptions in synaptic plasticity, oxidative stress, calcium signaling, etc., are also observed in AD patients^5^. Aging is the leading risk factor for AD, and most AD occurrences are sporadic. Numerous therapy strategies focused on the Aβ and tau-pathologies in recent decades have failed to demonstrate the desired efficacy for reducing cognitive deficits in AD patients. Several factors may contribute to these failures, including a lack of knowledge of the complicated multifactorial nature of AD, which may necessitate combination therapy over monotherapy, or to the fact that patients were treated after the disorder was already advanced^6^. Senile plaques and NFTs are the neuropathological hallmark of AD and may eventually lead to neuronal degeneration and death. Senile plaques predominantly form in the medial temporal lobe and a cortical layer of the brain’s hippocampus due to extracellular Aβ accumulation^7^. APP is sequentially processed by β-secretase and γ-secretase to produce Aβ. On a molecular level, β-secretase cleaves APP at its N-terminus, resulting in the membrane-bound APP fragment C99, which is then processed sequentially by β-secretase. This cleavage releases 42-amino-acid-long and 40-amino-acid-long fragments, designated Aβ42 and Aβ40, from the C99 N-terminus region into the extracellular space. Aβ42 enhances its abnormal aggregation, which results in neuronal death and brain amyloidosis because of its hydrophobic inclination. NFTs are another neuropathological feature of AD in addition to the Aβ42 filaments seen as senile plaques. In the weak neurons of twisted tau protein fibrils, NFTs build up. Tau is a microtubule-associated protein expressed mainly in the brain and whose physiological role is thought to stabilize the microtubules of neuronal cells^8^. The development of treatments that target Aβ and P-tau for AD treatment has received enormous effort, but these endeavors have largely failed. Finding new therapeutic approaches to control disorder progression is an urgent priority^9^. Scientific evidence suggests that alterations in neuronal autophagy may act as a prodegenerative trigger in AD. Abnormalities in this process will result in an abnormal and progressive buildup of intracellular and extracellular Aβ peptides in AD patients, which will facilitate the formation of Aβ plaques, which will activate microglia and cause the release of secondary proinflammatory cytokines, the production of reactive oxygen species (ROS), and the death of neuronal cells. Along with Aβ, the buildup of tau filaments and NFTs caused by hyperphosphorylated tau protein isoforms has also been linked to neuronal dysfunction after autophagy malfunction. Furthermore, polymorphisms linked to genes involved in the autophagy system have been found to significantly increase the chance of having a larger amyloid burden, according to genome-wide association analyses^10^. Autophagy is an internal breakdown and recycling process in eukaryotic cells that have been conserved throughout evolution. Numerous stressors, including nutritional scarcity, a lack of growth factors, an infection, and inflammation, trigger autophagy. Bulk autophagy, also known as nonselective breakdown and recycling of random cytoplasmic fraction, was once thought to result from nutrient or energy minimization stress. Removing specific undesired and dangerous cytoplasmic components, like protein aggregates and damaged organelles has been shown to play important cytoprotective roles through autophagy. This process is now termed selective autophagy^8^. Microautophagy, chaperone-mediated autophagy, and macroautophagy are the three kinds of autophagy based on how intracellular components are delivered into the lysosome for destruction. By directly invaginating the lysosomal membrane, the cytoplasmic material is taken up into the lysosome during microautophagy. Cytosolic proteins are more easily degraded thanks to chaperone-mediated autophagy, which sends them straight to lysosomes and into the lysosomal lumen. Degradable cytoplasmic components are enclosed in ‘autophagosomes,’ subcellular double-membrane structures, during macroautophagy. To be broken down, lysosomes receive the cell ‘waste’ from autophagosomes. The most common type of autophagy is macroautophagy^11^. When ubiquitin–proteasome systems and chaperones are overworked, and under stress, autophagy helps the body eliminate aggregated or improperly folded proteins. In general, autophagy is assumed to play a role in many human ailments, such as heart cancer and neurodegenerative disorders; however, autophagy dysfunction also plays a role in several protein aggregation disorders. Growing data suggests that misfolded proteins can reduce cell viability and function through various processes, including pore formation, proteasome suppression, and disruption of intracellular transport. It has been established that the ubiquitin–proteasome pathway and the molecular chaperone system cannot handle the generation of misfolded proteins. However, misfolded or aggregated protein is fiercely sequestered in a way that can be seen under a microscope, forming pericentriolar structures known as aggresomes. The aggresome–autophagy pathway is the process by which these aggresomes are subsequently destroyed. The function of autophagy in modulating aggresomes and their possible therapeutic activity in the control and management of AD focuses on many natural compounds that can potentially enhance AD therapy^12^. For neurons, in particular, the human nervous system primarily relies on autophagy to remove large, soluble protein clumps and preserve protein homeostasis. Additionally, neuronal cells are vulnerable to defective autophagy due to their special characteristics. For instance, because lysosomes are rarely found in distal axons, axonal autophagosomes in neurons must be brought to the cell body to combine with lysosomes^13^–^15^. Autophagy participates in the metabolism of Aβ, most likely through controlling its production and elimination. Aβ is created through the successive cleavage of the precursor protein APP by the enzymes β-secretase (BACE1) and γ-secretase. It has been shown that a substance-induced, autophagy-related-5-dependent autophagy promotes APP breakdown. Additionally, it was confirmed that APP and the four components of the γ-secretase complex originate in autophagosomes, indicating that at least some Aβ peptides are created via the autophagic pathway^16^,^17^. Along with Aβ metabolism, autophagy is crucial for the pathophysiology of tau. Initially, in-vitro results demonstrated that tau clearance was hampered after autophagic flow was inhibited, and insoluble tau clumps were extensively accumulated. Hyperphosphorylated tau is discovered to colocalize with the autophagosome marker LC3 and the autophagy receptor p62/SQSTM1 in the brains of familial AD patients, but this overlap is not seen in healthy controls^18^,^19^. Synaptic dysfunction is another defining aspect of AD pathogenesis, in addition to Aβ deposition and tau aggregation. Synapses are specialized neural structures that are the fundamental building blocks for communication between presynaptic and postsynaptic neurons. Synapse numbers have decreased during the early stages of AD pathogenesis. Recent research suggests that synaptic activities like neurotransmission and synaptic plasticity require functional autophagy^20^,^21^. The organelles responsible for generating energy are mitochondria. On the other hand, ROS are produced as byproducts of energy synthesis, and their buildup harms mitochondria. To preserve mitochondrial function and homeostasis, numerous quality control techniques have been found. Accumulated Aβ potently produces excessive ROS and extensively damages mitochondria throughout the development of AD. The primary method for removing damaged mitochondria is called mitophagy. Although gene therapy, immunotherapy, and other treatments are fascinating possibilities for treating AD, small compounds are still preferred because they can more easily penetrate the blood–brain barrier. Hundreds of substances have recently undergone clinical trials to treat AD^5^. Strategies for prospective AD treatment that target the autophagy–lysosomal pathway. Autophagosome production can be aided by targeting upstream autophagy signaling, such as activating AMPK with metformin, resveratrol, or berberine or inhibiting MTORC1 with rapamycin, everolimus, temsirolimus, or latrepirdine. Curcumin analog C1, Wy14643, cinnamic acid, and genocide XVII are a few small compounds that activate transcription factor EB. Lysosome functions are improved together with autophagy flux, which could be a viable anti-AD strategy. Isoproterenol, clenbuterol, and acid nanoparticles are all β-adrenergic agonists that can be used to enhance lysosomal activities by inhibiting the ClC-7 transporter directly. Importantly, it has been discovered that the small molecule autophagy enhancers indicated above help AD animal models with memory deficits by lowering Aβ or MAPT/tau aggregates^22^. The development of novel assays to detect dynamic autophagy flux in animals and humans is highly wanted both for animal models and clinical investigations because most medications can potentially have off-target effects. Thus, developing novel therapeutic techniques for treating AD may result from autophagy-targeted therapeutic approaches^11^,^22^.

## Ethical approval

This article does not require any human/animal subjects to acquire such approval.

## Patient consent

Not applicable.

## Source of funding

None.

## Author contributions

T.B.E.: conceptualization, writing – original draft preparation, writing – reviewing and editing, and visualization. H.C. and K.D.: data curation and writing – reviewing and editing.

## Conflicts of interest disclosure

The authors declare that they have no financial conflict of interest with regard to the content of this report.

## Research registration unique identifying number (UIN)

None.

## Guarantor

T.B. Emran, PhD, Associate Professor, Department of Pharmacy, BGC Trust University Bangladesh, Chittagong 4381, Bangladesh. Tel: +88 030 335 6193, Fax: +88 031 255 0224. https://orcid.org/0000-0003-3188-2272. E-mail: talhabmb@bgctub.ac.bd

## Declaration of competing interest

We have read and understood the policy on the declaration of interests and have no relevant interests to declare. The responsibility for the content lies with the author, and the views stated herein should not be taken to represent those of any organizations or groups with and for which he works.

## Provenance and peer review

Not commissioned, externally peer-reviewed.
